# Production of functional human nerve growth factor from the saliva of transgenic mice by using salivary glands as bioreactors

**DOI:** 10.1038/srep41270

**Published:** 2017-01-24

**Authors:** Fang Zeng, Zicong Li, Qingchun Zhu, Rui Dong, Chengcheng Zhao, Guoling Li, Guo Li, Wenchao Gao, Gelong Jiang, Enqin Zheng, Gengyuan Cai, Stefan Moisyadi, Johann Urschitz, Huaqiang Yang, Dewu Liu, Zhenfang Wu

**Affiliations:** 1 National Engineering Research Center for Breeding Swine Industry, College of Animal Science, South China Agricultural University, Guangzhou, 510642, China; 2 Guangdong Provincial Key Laboratory of Agro-animal Genomics and Molecular Breeding, College of Animal Science, South China Agricultural University, Guangzhou, 510642, China; 3 Institute for Biogenesis Research, Department of Anatomy, Biochemistry and Physiology, John A. Burns School of Medicine, University of Hawaii at Manoa, Honolulu, 96822, USA; 4 Manoa BioSciences, 1717 Mott-Smith Dr. #3213, Honolulu, 96822, USA

## Abstract

The salivary glands of animals have great potential to act as powerful bioreactors to produce human therapeutic proteins. Human nerve growth factor (hNGF) is an important pharmaceutical protein that is clinically effective in the treatment of many human neuronal and non-neuronal diseases. In this study, we generated 18 transgenic (TG) founder mice each carrying a salivary gland specific promoter-driven hNGF transgene. A TG mouse line secreting high levels of hNGF protein in its saliva (1.36 μg/mL) was selected. hNGF protein was successfully purified from the saliva of these TG mice and its identity was verified. The purified hNGF was highly functional as it displayed the ability to induce neuronal differentiation of PC12 cells. Furthermore, it strongly promoted proliferation of TF1 cells, above the levels observed with mouse NGF. Additionally, saliva collected from TG mice and containing unpurified hNGF was able to significantly enhance the growth of TF1 cells. This study not only provides a new and efficient approach for the synthesis of therapeutic hNGF but also supports the concept that salivary gland from TG animals is an efficient system for production of valuable foreign proteins.

Mammalian animals are highly efficient and low-cost platforms for the synthesis of high-quality human proteins with correct processing and post-translational modifications. Therefore, transgenic (TG) animals have been employed for the production of various therapeutically important human proteins^1^,^2^,^3^,^4^. To date, two therapeutic proteins produced from the milk of TG animals have been approved for commercial and clinical use in Europe and the USA^2^. Presently, mammary glands from TG animals are the most commonly used and promising bioreactors for pharmaceutical protein production, because they are able to efficiently synthesize and secrete high-level heterologous proteins into milk, which can be collected repeatedly by simple and innocuous methods for large-scale purification of target proteins^2^,^4^. However, the use of mammary glands as bioreactors has some disadvantages: (1) only TG female animals can produce heterologous proteins from their milk; (2) TG female animals can synthesize foreign proteins in their milk only when they are at the lactation stage; (3) in some animal species the lactation period is short and hence merely a small amount of transgene-encoded proteins can be produced from milk; (4) in addition, milk usually contains a large amount of diverse endogenous proteins, which complicates the isolation of high-purity foreign proteins. For example, human milk contains more than 1,600 proteins and has a total protein concentration of 13 mg/ml^5^, while cattle milk carries approximately 1,000 proteins and has 30 mg/ml of total proteins^6^. Hence, there is a need to develop new bioreactor systems for the efficient production of valuable proteins and here we report salivary glands as an excellent alternative.

Salivary glands are exocrine organs that naturally express and secret diverse biologically active proteins into the saliva^7^,^8^,^9^, which is continually produced by both male and female animals during their entire life span. Additionally, many species of animals secrete a large volume of saliva, often larger than the volume of milk produced. For example, pigs, goats, sheep and cows can produce an average of 15, 6–16, 6–16 and 60–190 liters of saliva per day respectively^10^,^11^,^12^. Furthermore, saliva contains a smaller number and amount of endogenous proteins than milk, which may be advantageous for the purification of expressed foreign proteins. For example, the number of proteins detected in the saliva of humans and cattle is about 1200^13^ and 900^13^ respectively, while the total protein concentration is 0.72 mg/ml^14^ and about 0.6–1.8 mg/ml^15^. More importantly, saliva can be collected repeatedly from various animals, including mouse, pig, cattle, sheep and goat by surgical or non-surgical methods^12^,^16^,^17^,^18^,^19^,^20^. The target proteins can then be isolated by large-scale purification. Taken together, these advantages suggest that salivary glands may serve as efficient bioreactors for protein production. To the best of our knowledge, however, there have been no reports on the successful production of proteins from the saliva of TG animals for use as pharmaceutical agents.

Previously, our group and others have described the generation of TG animals expressing microorganism-derived digestive enzymes such as phytase and cellulase specifically in the salivary glands for improvements of feed nutrient utilization^11^,^21^,^22^,^23^. Here we report the production of a therapeutic protein, the human nerve growth factor (hNGF), in the salivary glands of TG mice. Nerve growth factor (NGF) is a therapeutically important protein that was first identified by Cohen and Levi-Montalcini^24^,^25^. It not only is clinically relevant for the treatment of various neuronal ailments such as glaucoma and Alzheimer’s disease but also has promising therapeutic potential for some non-neuronal disorders such as vascular and immune diseases^26^,^27^,^28^,^29^,^30^. Commercial mouse NGF (mNGF) that is purified from mouse submandibular glands has been approved in China for the treatment of some nerve damage and degeneration diseases, including optic nerve injury, spinal cord injury, traumatic brain injury, Alzheimer’s disease, Parkinson’s disease, hypoxic-ischemic encephalopathy and pediatric cerebral palsy in humans. Currently, the cost of therapeutic mNGF in China is approximates $1500 per milligram and the total amount of sales for mNGF in the Chinese market reached 500 million US dollars in 2016. However, mNGF and hNGF are remarkably different in their biological and biochemical properties, as a recent study clearly indicated that mNGF not only shows higher sensitivity to proteolytic cleavage, chemical and thermal denaturation but also exhibits significantly weaker bioactivity than hNGF in human cells^31^. Furthermore, administration of mNGF to humans may induce immunogenic responses to this exogenous protein in patients.

To address these concerns, hNGF has been produced in *E. coli*^32^,^33^, yeast^34^, insect cells^35^,^36^,^37^,^38^ and mammalian cells^39^,^40^,^41^. Yet in these cell systems the yield of the hNGF protein is low, and some of them, such as the *E. coli* and the yeast systems might be unable to provide correct post-translational modifications for hNGF. To increase the yield of hNGF, Coulibaly *et al*. have used the mammary gland of TG rabbits as an alternative system to synthesize functional hNGF^42^. However, mammalian salivary glands might be better suited for expression of hNGF, since biologically active host NGF is naturally expressed in the salivary glands of humans and mice^43^,^44^,^45^,^46^, suggesting that salivary glands can provide processing and modifications for the correct assembly of NGF.

To test the feasibility of utilizing salivary glands of TG animals as efficient bioreactors for the synthesis of therapeutically important hNGF, we generated TG mice that specifically expressed hNGF in their salivary glands, purified the secreted hNGF from their saliva and characterized the function as well as the bioactivity of purified hNGF.

## Results

### Production and identification of TG founder mice

**Figure 1:**
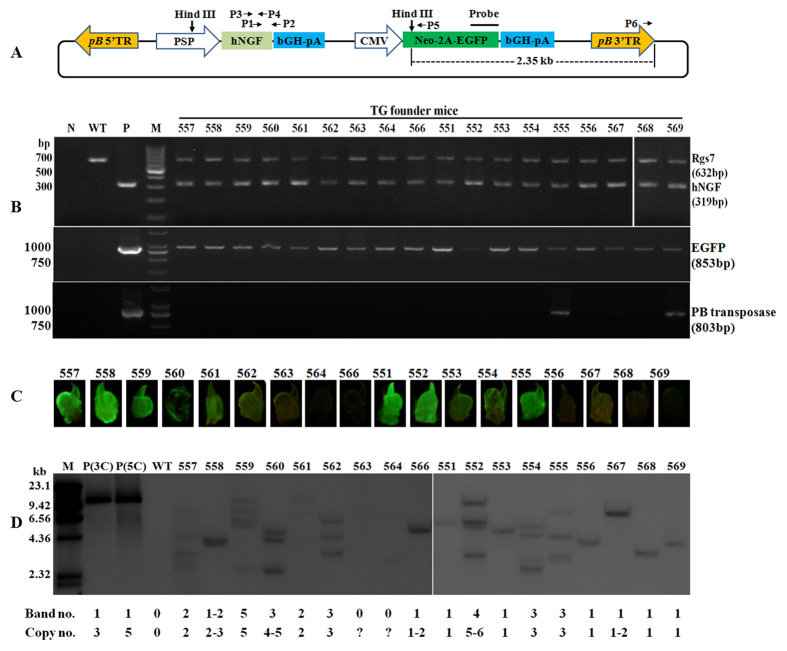
Production and identification of TG founder mice. (**A**) Structure of the salivary gland-specific human NGF (hNGF) plasmid pmPSP-hNGF. The transposon is flanked by the *pB* 5′TRE and *pB* 3′TRE, the *piggyBac* transposon 5′ and 3′terminal repeat elements. It was assembled to contain (5′ to 3′): PSP, the mouse parotid secretory protein gene promoter, which is salivary glands specific; the hNGF gene; the bovine growth hormone gene poly-A signal (bGH-pA); the cytomegalovirus promoter (CMV) driving the Neomycin-resistance gene and EGFP gene, linked by a 2 A peptide (Neo-2A-EGFP); and finally a bGH-pA. The location of primer set #1 (P1 + P2), #2 (P3 + P4), and #3 (P5 + P6), which were used for PCR, qPCR/RT-PCR and inverse PCR respectively, as well as the probe and enzyme used for Southern blot are also shown on the plasmid map. (**B**) PCR identification of TG F_0_ founder mice. N represents negative control using water as template, P positive control using plasmid pmPSP-hNGF or pm*PB* as template, M represents molecular markers and Rgs7 is for the regulator of G protein signaling 7, which was used as an internal control gene. (**C**) EGFP expression in the claw tissues of TG F_0_ mice. (**D**) Analysis of transgene integration patterns in the genome of TG F_0_ mice by Southern blot. M depicts molecular markers. P (3 C) and P (5 C) are samples where three copies (22.3 pg) or five copies (37.2 pg) of the plasmid were added to 10 μg of WT mouse genomic DNA as positive controls. The absence of a positive signal for 563 and 564 TG F_0_ mice could be due to degradation of their genomic DNAs as their samples were isolated from the postmortal tail tissues, while all other genomic DNA samples were extracted from live mice’s tail biopsies.

A pmPSP-hNGF donor plasmid, harboring a *piggyBac* transposon that carries the expression cassettes for a salivary glands-specific hNGF transgene and a selectable marker gene (Neo-2A-EGFP) was successfully constructed (Fig. 1A). The pmPSP-hNGF plasmid was co-injected with the *PB* transposase expression helper plasmid pm*PB*^47^ into the pronuclei of 96 mouse zygotes. Following transfer of 90 microinjected embryos into the oviducts of surrogate females, 35 pups were born and 18 of them were identified as TG founder (F_0_) mice as the hNGF transgene and the EGFP marker gene were detected in their genomic DNA by PCR (Fig. 1B and Table 1). Two TG founder mice, 555 and 569, also carried the pm*PB* helper plasmid-derived *PB* transposase gene (Fig. 1B), which might cause re-transposition of inserted *PB* transposon harboring the hNGF transgene. TG founders expressed various levels of EGFP (Fig. 1C) and no abnormal phenotype was observed on any of the TG F_0_ mice.

To investigate the transgene integration patterns in TG F_0_ mice, genomic DNA of all TG founders was analyzed by Southern blot. The results depicted in Fig. 1D indicated that the transgene was inserted in a monogenic manner, with the copy number varying from 1 to 6.

### Selection of TG mouse line producing the highest level of hNGF in the saliva

**Figure 2:**
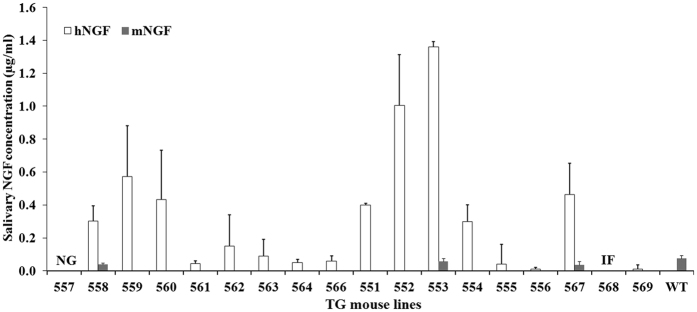
Average salivary hNGF concentrations among different TG mouse lines as detected by ELISA analysis. Three to six 30-day-old TG F1 mice from each line were randomly selected for analysis. Endogenous salivary mNGF concentration also was measured by ELISA for mice of some of the lines. Each value is present as Mean ± SEM. NG means no germline transmission of transgene in TG founder 557. IF represents infertile TG founder 568.

In order to identify the TG mouse line expressing the highest level of hNGF protein in its saliva, 3–6 TG F_1_ generation mice, produced by the breeding of TG F_0_ individuals to wild type (WT) individuals, were randomly selected from each F_1_ line and their average salivary hNGF concentration was determined by ELISA. The results demonstrated that TG F_1_ progenies of line 553 secreted the highest level of hNGF (1.36 ± 0.06 μg/mL) into their saliva (Fig. 2). Interestingly, we noticed that F1 animals from lines 552, 559, and 560, whose founders carried multiple transgenes copies, showed large variations in secreted hNGF concentrations. In contrast, F_1_ mice from lines 551 and 553, whose founders carried only one transgene copy displayed small concentration variations (see error bars in Fig. 2). The large variation in salivary hNGF concentration observed on F_1_ mice from lines 552, 559 and 560, might be due to the difference in transgene copy numbers by different TG F_1_ progenies from the same line founder. This might have resulted from segregation of the multiple copies of monogenically inserted transgenes after their transmission from the same line founder to its TG F_1_ progenies. Furthermore, we observed that two TG founders (551 and 553) with a single copy of transgene, passed this transgene to about 50% of their F_1_ offspring, while nearly 90% of F_1_ progenies inherited the transgene from two TG founders (559 and 560) carrying multiple copies of transgenes (Table 2). The high percentage of transgenic F_1_ offspring observed in lines 559 and 560 could also have resulted from segregation of the multiple copies of monogenically inserted transgenes during their transmission from the F_0_ founders to the TG F_1_ mice.

The hNGF-expressing TG mice also secreted a low level (0.04–0.06 μg/ml) of endogenous mNGF into their saliva (Fig. 2). A similar level (0.07 ± 0.02 μg/ml) of mNGF was also detected in the saliva of WT mice (Fig. 2).

We chose line 553 TG mice and their WT littermates for subsequent investigation, as F_1_ TG mice from this line produced the highest level of hNGF in their saliva and with the lowest variation among the individual TG mice.

### Identification of transgene integration site in the genome of TG F_0_ founder mouse of line 553

**Figure 3:**
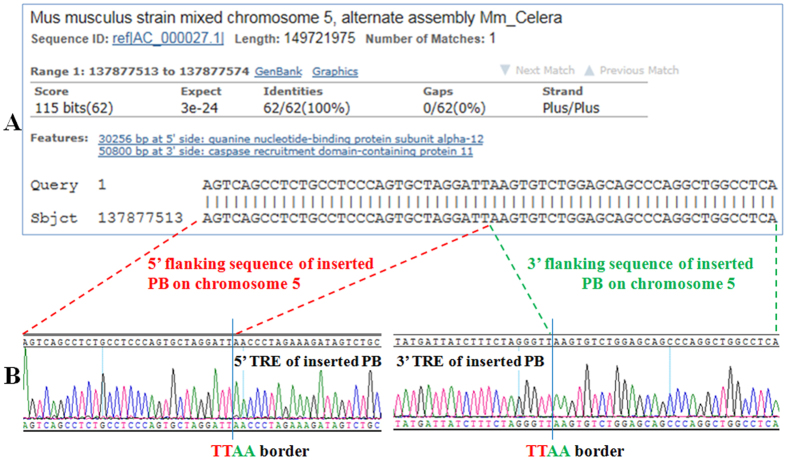
Identification of the transgene insertion site in the genome of TG founder 553. (**A**) Blast result of transgene insertion site. “Query” represents the genomic sequence flanking the *PB* transposon in TG founder 553 (see **B**). “Sbjct” represents the part of the reference sequence of mouse chromosome 5 that matches the chromosomal sequence flanking the *PB* transposon; (**B**) The sequencing results of the inverse PCR product, which shows the 5′ and 3′terminal repeat element (TRE) sequences of the inserted *PB* transposon and the chromosomal sequence flanking the inserted *PB* transposon.

To determine the insertion site of the single copy of hNGF transgene in the genome of TG mice of line 553, the genomic DNA of TG founder 553 was analyzed by inverse PCR. The results (Fig. 3) demonstrated that the transgene was inserted into the noncoding intergenic sequence between the guanine nucleotide-binding protein subunit alpha-12 gene and the caspase recruitment domain-containing protein11 gene on chromosome 5. The results (Fig. 3) also indicated that hNGF integration had been mediated by *PB* transposition as the transgene was flanked by TTAA sequences, the recognition sequence for the *PB* transposase.

### Characterization of transgene expression patterns in TG F_1_ mice of line 553

**Figure 4:**
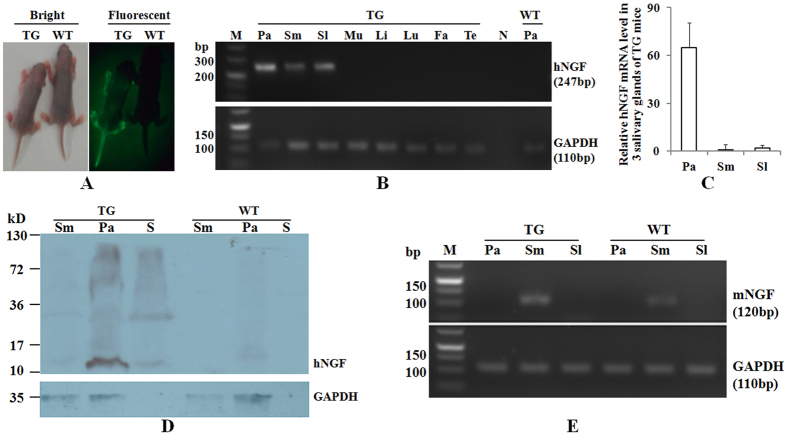
Characterization of transgene expression in TG F_1_ mice of line 553. (**A**) Analysis of EGFP expression in TG mice. (**B**) Analysis of hNGF mRNA expression in different tissues of TG mice by RT-PCR. (**C**) Analysis of relative hNGF mRNA expression level in 3 salivary glands of TG mice by qPCR. Relative hNGF mRNA levels were normalized to the hNGF transcription levels in the submandibular gland (Sm), which was defined as 1. (**D**) Analysis of hNGF protein expression in TG and WT mice by Western blot. (**E**) Analysis of endogenous mNGF mRNA transcription in 3 salivary glands of TG mice and their WT littermates by RT-PCR. Three TG F_1_ mice were analyzed in (**A,B** and **E**), and all of them showed similar results, hence only a representative result is shown in (**A**,**B** and **E**). Results in (**C**) was derived from the analysis of pooled mRNA samples of four (2 males + 2 females) 30-day-old TG F_1_ mice, while results in (**D**) were derived from the analysis of pooled total protein samples of four (2 males + 2 females) 30-day-old TG or WT F_1_ mice. Pa, parotid gland, Sm, submandibular gland, Sl, sublingual gland. Mu-muscle, Li-liver, Lu-lung, Fa-fat, Te-testis, N-negative control using water as template, S-saliva.

All TG F_1_ mice of line 553 showed strong EGFP expression as demonstrated by epifluorescence (Fig. 4A), indicating a stable transmission of the transgene from the 553 founder to its progenies. To analyze tissue specificity of hNGF transgene expression in TG mice, 8 different tissues collected from TG F_1_ mice of line 553 were analyzed by RT-PCR. The results confirmed that hNGF is specifically expressed in 3 salivary glands, including parotid, submandibular and sublingual glands, but not in muscle, liver, lung, fat and testis of TG mice (Fig. 4B). Although hNGF mRNA was detected in all three salivary glands, parotid glands contained higher levels than submandibular and sublingual glands (Fig. 4C). Western blot results (Fig. 4D) indicated that mature hNGF protein is mainly expressed in the parotid glands but not in the submandibular glands, which is consistent with the hNGF mRNA expression patterns found in the TG mice (Fig. 4C). Mature hNGF was also detected in the saliva of TG mice (Fig. 4D), suggesting it is successfully secreted from salivary glands into the saliva. Similar to their WT littermates, TG mice also express endogenous mNGF uniquely in the submandibular glands (Fig. 4E), which is consistent with previously reported results^43^.

### Purification of hNGF from the saliva of TG F_1_ and F_2_ generation mice of line 553

**Figure 5:**
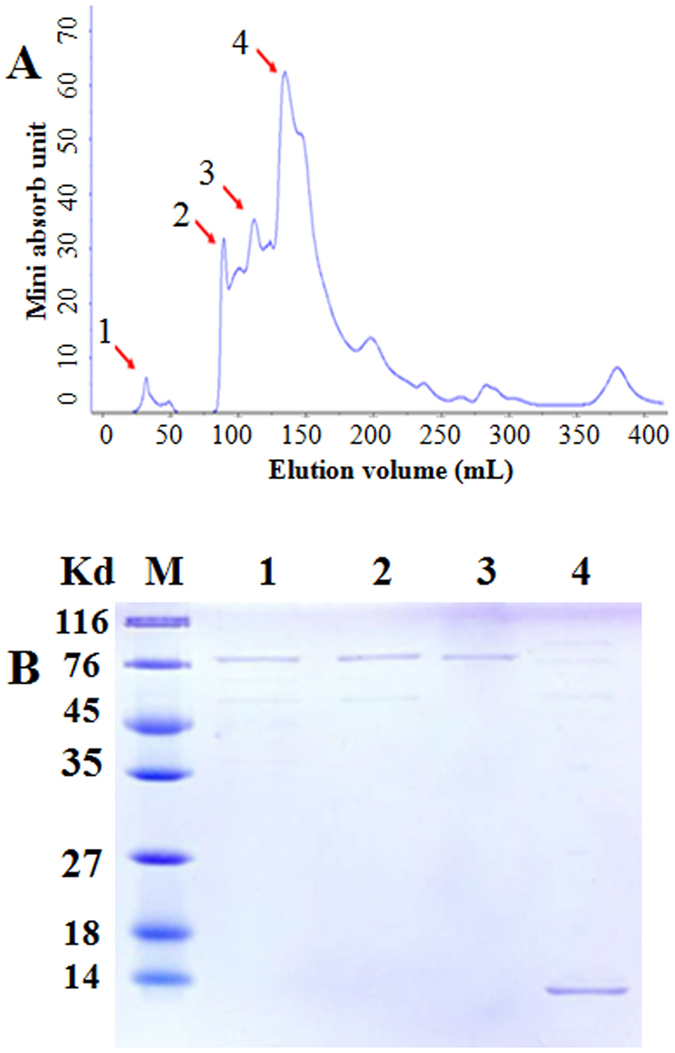
Purification of hNGF from the saliva of TG F_1_ and F_2_ mice of line 553. (**A**) Analysis of 4 eluted protein fractions (#1–4) by UV absorption after passing the saliva through the purification column. (**B**) Analysis of eluted protein fractions (#1–4) by SDS-PAGE. Only the #4 eluted fraction contains a protein with a molecular weight of 13.5 kD which matches the molecular weight of mature hNGF.

A protein with a molecular weight of 13.5 kD that matches the molecular weight of mature hNGF was purified, by size-exclusion chromatography-based from saliva collected from TG F_1_ and F_2_ mice of line 553 (Fig. 5). Approximately 28 μg of hNGF was purified from about 40 mL of saliva, resulting in a yield of 51.47% (=28 μg/40 mL × 1.36 μg/mL).

### Identification of purified hNGF

**Figure 6:**
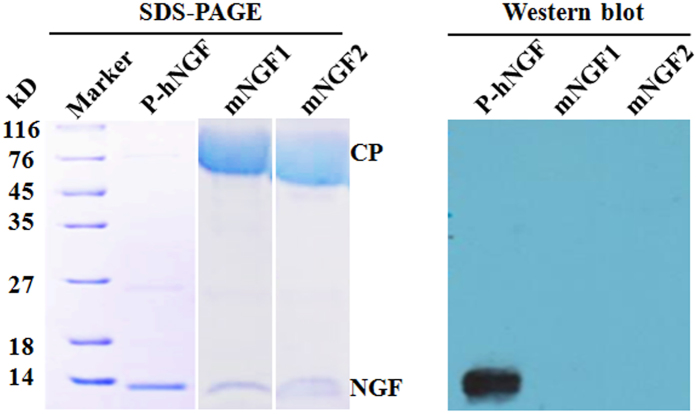
Identification of purified hNGF (P-hNGF) by SDS-PAGE and Western blot analysis. mNGF1 (Staidson, Beijing, China) is the mouse NGF that was isolated from mouse submandibular glands and is currently an approved human drug for sale in China. mNGF2 (Cat. #1156-NG, R & D systems, Minneapolis, MN, USA) is the mouse NGF that was expressed and purified from mouse myeloma cells. CP represents carrier protein, which is human serum albumin (66.4 kD) for mNGF1 and bovine serum albumin (66.4 kD) for mNGF2. The molecular weight of purified hNGF is 13.5 kD.

**Figure 7:**
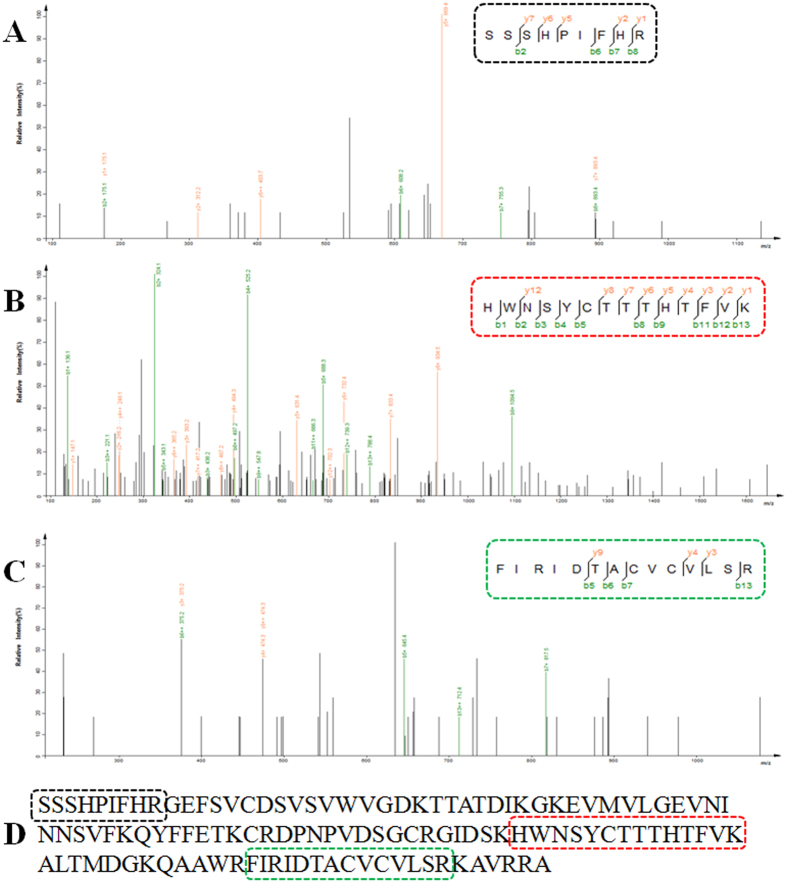
Identification of the amino acid sequence of hNGF purified from the saliva of TG mice of line 553 by liquid chromatography-mass spectrum/mass spectrum (LC-MS/MS) analysis. (**A–C)** LC-MS/MS analysis of 3 different short peptides derived from trypsin digestion of purified hNGF. Each short peptide’s amino acid sequence that was identified by LC-MS/MS is shown inside the frame in the right upper corner of each panel. (**D**) The amino acid sequence of 3 LC-MS/MS-identified short peptides (inside the black, red and green frame) and their position on the amino acid sequence of mature hNGF protein.

Like mNGF, hNGF purified from the saliva of line 553 TG mice had a molecular weight of 13.5 kD, and showed reactivity with the anti-hNGF monoclonal antibody which was not reactive with mNGF in Western blots (Fig. 6). Partial amino acid sequences of purified hNGF were verified by liquid chromatography-mass spectrum/mass spectrum (LC-MS/MS). The verified amino acid sequences matched the corresponding amino acid sequences of mature hNGF (Fig. 7), confirming the 13.5 kD protein isolated from the saliva of TG mice as being hNGF.

### Function and bioactivity of hNGF purified from TG mice’s saliva

**Figure 8:**
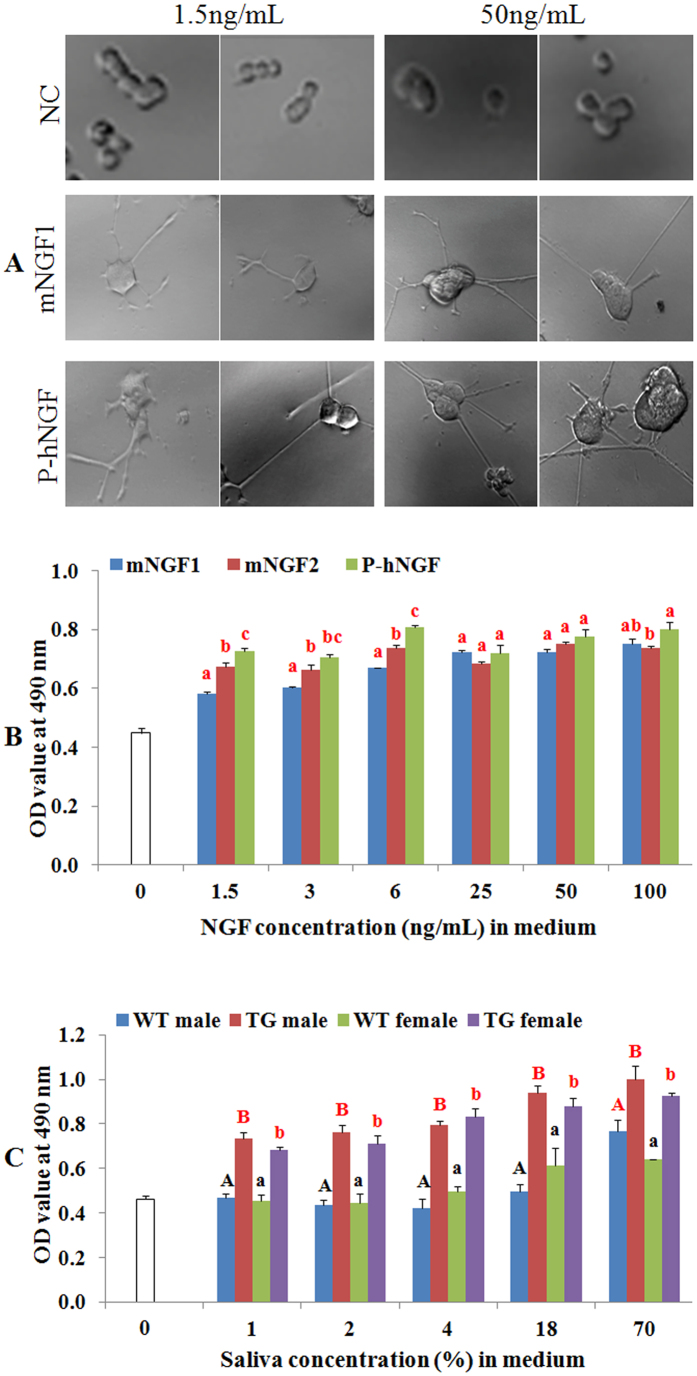
Bioassay of purified hNGF (P-hNGF) and saliva collected from TG mice. (**A**) Analysis of the activity of P-hNGF on neuronal differentiation of PC12 cells. NC represents negative control while mNGF1 (Staidson, Beijing, China) was isolated from mouse submandibular glands and is currently an approved human drug for sale in China. (**B**) Comparison of the activity on human TF1 cells proliferation between commercial mNGFs and purified hNGF. mNGF2 (Cat. #1156-NG, R & D systems, Minneapolis, MN, USA) was expressed and purified from mouse myeloma cells. The number of TF1 cells is positively correlated with the OD value measured at 490 nm. Values of a same concentration group labeled with different letters are statistically different at P < 0.05. (**C**) Comparison of the activity on human TF1 cell proliferation between line 553 TG mice’s saliva and WT mice’s saliva. Values of a same concentration group labeled with different letters are statistically different at P < 0.05. Values labeled with red letters are statistically different from that of the 0 concentration group at P < 0.05.

The hNGF protein purified from the saliva of line 553 TG mice was highly functional in inducing neuronal differentiation of PC12 cells, as neurite formation was observed at low levels (1.5 ng/mL) of purified hNGF (Fig. 8A). Purified hNGF was also very efficient in promoting proliferation of the TrKA receptor-carrying TF1 cells (Fig. 8B). In addition, the bioactivity of purified hNGF was higher than that of mNGF, especially at low concentration (Fig. 8B). Surprisingly, even saliva from TG mice could significantly enhance TF1 cell growth in a dose-dependent manner, and its capacity of promoting TF1 cell proliferation was significantly (P < 0.05) higher than the saliva of WT mice (Fig. 8C).

## Discussion

In this set of experiments, we have used mouse salivary glands as bioreactors to successfully produce highly functional and active hNGF. The production rate of salivary hNGF (13.5 kD) in TG mice of line 553 reached 1.36 μg/ml or 0.10 μmol/ml. A previous study^11^ reported that TG mice carrying 3 copies of mouse PSP promoter-controlled phytase transgene synthesized 15 μg/ml of the 55 kD salivary phytase, an enzyme that increases dietary phosphorus utilization. These values translate into an average of 0.09 μmol/l of phytase per copy of the transgene. Such protein synthesis efficiency is very similar to that found in our line 553 TG mice carrying only one copy of the hNGF transgene. These data suggest that the protein expression level of a single copy of salivary gland-specific mouse PSP promoter-controlled transgene in the saliva of TG mice is generally at about 0.09–0.1 μmol/ml, if it is integrated into a transcriptionally active genomic site.

In this study, TG mice were generated by *piggyBac* transposition-mediated gene transfer. This transgenesis method was highly efficient as 20% of embryos injected with *piggyBac* plasmids developed into TG F_0_ pups carrying the hNGF transgene. As observed in this and earlier studies^48^,^49^, TG animals produced by this method usually carry only one and up to five monogenic transgene copies.

Although the data from this study showed no direct correlation between transgene copy number and hNGF expression levels in the saliva of TG mice, we observed that TG mice with multiple transgene copies, including lines 552, 558, 559, and 560 (see Fig. 1D) had higher hNGF concentration in their saliva (see Fig. 2). Previous studies have indicated that the transgene copy number is positively correlated with expression level in TG animals^50^,^51^. Therefore, it may be possible to increase the production level of hNGF by generating TG animals with higher transgene copy numbers, for example by increasing the amount of plasmid DNA used during microinjection^48^. Transgene copy number can also be doubled by producing TG homozygotes simply by mating heterozygous TG animals. In addition, use of a stronger salivary gland-specific regulatory element, such as the proline-rich protein R15 promoter^11^ for controlling transgene expression might also be able to improve the synthesis rate of heterologous proteins in the saliva of TG animals.

Our Western blot (Fig. 4D) probed with anti-hNGF antibody showed, in addition to the expected 13.5 kD band, a ~30 kD band in the saliva, parotid gland, and submandibular gland samples of TG mice. This band could represent either the post-translationally modified mature form of hNGF or precursor forms of hNGF, including pro-hNGF and pre-pro-hNGF, or the complexes formed by them and other molecules. Although we did not determine the identity of this ~30 kD protein, our results were similar to previous studies which also reported a strong ~30 kD anti-hNGF antibody-reactive band in normal human oral mucosa^52^ and healthy human saliva^53^. These results suggest that the ~30 kD protein is a natural common product generated during *in vivo* processing and synthesis of hNGF.

The mouse PSP promoter used in this study has been shown to drive high-level transgene expression in the salivary glands of pigs^22^, which are able to secrete a large volume of saliva with an average of 15 L/day/adult pig^11^. Following the methods used for saliva collection in sheep and cattle^18^,^19^, we have recently developed a procedure based on cannulation of the parotid duct for long-term and large-volume saliva collection in pigs. By using this technique, we have successfully collected an average 3 L of saliva/day from adult pigs, which accounts for 20% of the total saliva produced, without causing any obvious injurious effects on them during a 40-day-long trial (unpublished data). With the establishment of this saliva collection technique, large-scale production of pharmaceutical proteins from the saliva of TG pigs may become feasible. If an adult TG pig produces similar levels of salivary hNGF as detected in line 553 TG mice (1.36 μg/mL), it would synthesize about 7.5 g of hNGF per year in its saliva, and at least 20% of secreted hNGF could be recovered from the saliva of TG pigs by using the saliva collection technique that we have established.

In our study hNGF was mainly expressed as the mature protein (13.5 kD) rather than the pro-hNGF (25 kD) in the salivary glands of TG mice. The mature hNGF protein that we purified from the saliva of TG mice not only had the ability to induce neuronal differentiation of PC12 cells but also strongly promoted proliferation of TF1 cells. Similar to the results reported by a previous study^31^, the hNGF produced in the present study also showed higher activity than mNGF in enhancing proliferation of human TF1 cells. These results indicate that the hNGF protein expressed in the salivary glands of TG mice was properly processed, modified, and secreted. In addition to NGF, many other physiologically and clinically important proteins are naturally synthesized in the salivary glands and secreted into the saliva of mammals^7^,^8^,^9^. Therefore, salivary glands may serve as ideal sites for the expression of pharmaceutically valuable proteins.

In summary, we have confirmed the feasibility of using salivary glands from TG animals as bioreactors for the synthesis of foreign proteins and demonstrated efficient production of highly functional and active hNGF protein from the saliva of TG mice. Additionally, with the availability of techniques for long-term collection of large volumes of saliva from various livestock salivary gland of TG animals may provide an attractive system for production of therapeutic proteins.

## Materials and Methods

### Ethics statement

This study was carried out in strict accordance with “The Instructive Notions with Respect to Caring for Laboratory Animals”, issued by the Ministry of Science and Technology of China. The animal experimental protocol was approved by the Institutional Animal Care and Use Committee of South China Agricultural University. All efforts were made to minimize animal suffering.

### Plasmid construction

The 12.6 kb mouse parotid secretory protein (PSP) gene promoter, starting at 11.75 kb upstream of transcription site to 0.84 kb downstream of transcription site^11^,^54^ was PCR amplified from mouse genomic DNA. A 771 bp recombinant human NGF coding sequence (CDS) was synthesized by the Genewiz company (Suzhou, China). This CDS was generated by replacing the 54 bp signal peptide in the 765 bp CDS of the human NGF gene (GenBank accession number: NM_002506.2), with a 60 bp mouse PSP signal peptide (GenBank accession number: X01697.1). The *piggyBac* 5′ and 3′terminal repeat elements and a fragment containing bGH polyA-CMV promoter-Neo-2A-EGFP- bGH polyA (see Fig. 1A) were also synthesized and ligated with the mouse PSP promoter and the recombinant human NGF CDS. The ligated fragment was then used to replace the fragment between the XhoI and Not I sites in pGEM-T plasmid (Promega, Madison, WI, USA), to generate the 20.1 kb pmPSP-hNGF plasmid. The DNA sequences of pmPSP-hNGF plasmid were confirmed by sequencing.

### Microinjection

The pmPSP-hNGF plasmid (9 ng/μl) was mixed at a volume ratio of 1:1 with the *piggyBac* transposase expression helper plasmid pm*PB* (3 ng/μl), a kind gift from The Wellcome Trust, Sanger Institute (Cambridgeshire, UK) and was constructed as previously described^47^. A mixture of the two plasmids was microinjected into the pronuclei of C57BL/6 mouse one-cell embryos, which were then transferred into the oviducts of ICR strain surrogate females. The surrogate females were mated with vasectomized males of the same strain on the day before embryo transfer. Pregnant females were allowed to deliver and raise their pups.

### PCR analysis

Genomic DNA was isolated from tail biopsies of TG F_0_ founder mice using a Tissue DNA extraction kit (Omega, Doraville, GA, USA). Primer set #1 (P1 + P2, for primer location, see Fig. 1A, for primer sequences see Table 3) was used to amplify the hNGF transgene. The EGFP marker gene, *PB* transpose gene and the internal control gene of Rgs7 were also amplified by PCR (for primer sequences see Table 3). The PCR amplification products were sequenced to confirm their identities.

### Observation of EGFP expression

EGFP expression in the claw tissues of TG F_0_ mice was analyzed by fluorescence microscopy. EGFP expression by epifluorescence in new born TG F_1_ mice was visualized by the Living Organism’s Fluorescent Protein Observation System (Model: FBL/Basic-B & N-01, BLS ltd., Budapest, Hungary). Photographs of TG and WT F_1_ mice were taken under blue light or normal light by a camera equipped with and without light filters.

### Southern blot analysis

Ten micrograms of tail genomic DNA from each TG F_0_ mouse was digested with Hind III and separated by electrophoresis in a 0.8% agarose gel. The DNA was subsequently transferred to a nylon membrane (GE Healthcare Life Sciences, Shanghai, China) by the capillary transfer method. The membrane was then prehybridized overnight at 42 °C and then hybridized with an 800 bp EGFP gene probe labeled with digoxigenin (DIG) by using a PCR DIG Probe Synthesis Kit (Roche Applied Science, China). Hybridization and wash steps were performed according to the manufacturer’s protocol using the DIG-High Prime DNA Labeling and Detection Starter Kit (Roche Applied Science, China). After hybridization, the membrane was incubated for 30 min in blocking solution and subsequently incubated for a further 30 min in Anti-Digoxigenin-AP antibody solution. The membrane was then incubated with 1 mL of CSPD ready-to-use solution for 5–20 min, and a Southern blot photograph was captured with an EC3 imaging system (UAP, CA, USA). Location of Hind III enzyme sites and EGFP probe on the pmPSP-hNGF plasmid are indicated in Fig. 1A.

### Saliva collection

Mice were anesthetized by intraperitoneal injection with anesthetic consisting of ketamine (25 μg per gram of body weight) and xylazine (1.1 μg per gram of body weight). Following anesthetization, mice were injected with 100 μg/mL pilocarpine hydrochloride (0.5 μg per gram of body weight) for stimulation of saliva secretion. About 100–200 μl of saliva were collected from the mouth of each mouse by pipetting in approximately 20 minutes as previously described^55^. Collected saliva was stored at −80 °C for later use.

### Enzyme-linked immunosorbent assay (ELISA) analysis

Salivary hNGF concentration was measured by the ELISA Kit for hNGF (Cat. No. E0105h, EIAab Science, Wuhan, China), and salivary mNGF concentration was measured by the ELISA Kit for mNGF (Cat. No. E0105m, EIAab Science, Wuhan, China), following the operating instructions provided with the kits.

### Inverse PCR analysis

One microgram of genomic DNA extracted from TG F_0_ mouse 553 was digested with Hind III. The digestion product was purified by a DNA purification column (Qiagen, Hiden, Germany) and eluted with 100 μL of ddH_2_O. After adjustment with ddH_2_O and T4 ligase buffer to a final required volume, T4 ligase was added with a final concentration of 10 U/μL in a 1000 μL final ligation mixture. The ligation reaction was allowed to proceed overnight by incubation at 16 °C and ligated DNA was purified via a Qiagen DNA purification column. After elution from the column with 100 μL of ddH_2_O, a 2 μL elution aliquot was used as template for the PCR reaction with primer set #3 (P5 + P6, for primer location see Fig. 1A, for primer sequences see Table 1). The resulting PCR products were cloned by ligation into a TA vector (Life Technologies, Carlsbad, CA, USA) and sequenced. The sequencing results were analyzed to obtain the genomic sequences flanking the inserted *PB* transposon, which were used to blast against the Mus musculus (house mouse) genomic DNA sequence database on NCBI BLAST website to find out the integration sites of the inserted *PB* transposon.

### Quantitative PCR (qPCR) and reverse transcription PCR (RT-PCR) analysis

Total RNA was extracted from different tissues of TG F_1_ mice of line 553 by E.Z.N.A Total RNA Kit I (Omega Bio-tek, Doraville, GA, USA). cDNA was synthesized by PrimeScript RT reagent Kit with gDNA Eraser (TaKaRa, Dalian, China). Primer set #2 (P3 + P4, for primer location see Fig. 1A, for primer sequences see Table 3) was used to amplify the cDNA of hNGF transgene. The cDNAs of endogenous mNGF gene and the internal control GAPDH gene were also amplified (for primer sequences see Table 3). The reverse transcription PCR (RT-PCR) was run by a PCR instrument (Bio-Rad, Hercules, CA, USA). The quantitative PCR (qPCR) was performed by the Eco Real-time PCR system (Illumina, San Diego, CA, USA) using SYBR Premix Ex Taq (TaKaRa, Dalian, China). The relative transgene expression level was calculated by the 2^−ΔΔCt^ method. The RT-PCR and the qPCR products were sequenced to confirm their identities.

### SDS-PAGE and Western blot analysis

The concentration of extracted tissue protein and salivary protein of each sample was determined by a Bradford assay method. A same amount of total protein for TG samples and their WT counterparts was loaded into a 10% SDS-PAGE gel and fractionated. Proteins were subsequently transferred to a PVDF membrane by the Trans-Blot SD Semi-Dry Electrophoretic Transfer Cell instrument (Bio-Rad, Hercules, CA, USA). Detection of hNGF on the membrane was carried out by using monoclonal rat anti-hNGF primary antibody (Cat. #MAB2562, R & D systems, Minneapolis, MN, USA), HRP-conjugated goat anti-Rat secondary antibody (Jackson Immuno Research, West Grove, PA, USA), and SuperSignal West Pico Chemiluminent Substrates (Thermo Scientific Pierce, Guangzhou, China) following the manufacturer’s protocols. mNGF1 (Staidson, Beijing, China) which was isolated from mouse submandibular glands and is currently an approved human drug for sale in China, and mNGF2 (Cat. #1156-NG, R & D systems, Minneapolis, MN, USA) which was expressed and purified from mouse myeloma cells were used as controls.

### Purification of hNGF

About 40 mL of saliva was collected from 80 TG F_1_ and F_2_ mice of line 553 at the age of 30 days following the method described above. Saliva was collected 3–4 times from each TG mouse. The mixed saliva was concentrated to 500 μl by 3 kD cut-off ultrafiltration tubes and then applied to a HiPrep 26/60 Sephacryl S-100 High Resolution column (GE Healthcare, Little Chalfont, UK) connected to the AKAT protein purification system (GE Healthcare, Little Chalfont, UK). Each eluted protein fraction (about 100 μl) was monitored by UV absorption at 280 nm, and was collected for SDS-PAGE and Western blot analysis. Fractions containing the 13.5 kD anti-hNGF antibody-reactive protein were mixed and the hNGF protein concentration was determined by ELISA as described above.

### Liquid chromatography-mass spectrum/mass spectrum (LC-MS/MS) analysis

The amino acid sequence of purified hNGF was analyzed by LC-MS/MS method as previously reported^56^. Briefly, purified hNGF was analyzed by electrophoresis in a polyacrylamide gel. After staining with Coomassie the 13.5 kD hNGF protein band was cut out from the gel and macerated into small cubes, destained by ammonium bicarbonate and acetonitrile, and digested with trypsin for 10 h. The digested products were then analyzed by the LC-MS/MS system (Eksigentekspert nanoLC and TripleTOF5600-plus, AB Sciex, Framingham, MA, USA) for verifying the presence of hNGF.

### PC12 cell differentiation assay

Undifferentiated rat PC12 cells were cultured for 4 days in Roswell Park Memorial Institute (RPMI) 1640 medium containing 1% fetal bovine serum (FBS) and 1% horse serum. Cells then were washed and resuspended in the wells (1 × 10^4^ cells/well) of collagen coated 6-well plates. Purified hNGF and mNGF1 (Staidson, Beijing, China) which was isolated from mouse submandibular glands and is currently an approved human drug for sale in China, were added to the wells on days 1 and 3. Morphology of PC12 cells were examined under an inverted microscope and photographed on day 6.

### TF1 cell proliferation assay

Human TF1 cells (Sigma-Alrich) were cultured for 1 week in RPMI-1640 medium containing 10% FBS with 2 ng/mL rhGM-CSF (R & D System, Minneapolis, MN, USA). Cells were then washed and resuspended in RPMI-1640 + 10% FBS to a concentration of 420,000 cells/mL and replated on 96-well microplates (12,600 cells per well in 30 mL). To compare the effects on TF1 cells proliferation between commercial mNGFs, including mNGF1 (Staidson, Beijing, China) and mNGF2 (Cat. #1156-NG, R & D systems), and purified hNGF, medium was removed 1 h after TF1 cells replating, and the wells were respectively added with 100 μL of RPMI-1640 containing 10% FBS and 0, 1.5, 3, 6, 25, 50 and 100 ng/mL of mNGF or purified hNGF. To compare the effects on TF1 cells proliferation between TG mice’s saliva and their WT littermate’s saliva, medium was removed 1 h after TF1 cells replating, and the cells of each group were respectively added with 100 μL of RPMI-1640 containing 10% FBS and 0%, 1%, 2%, 4%, 18%, and 70% of mixed saliva collected from 3–5 mice from each group of line 553. Each treatment was performed in duplicate. After a 40 h incubation period at 37 °C and 5% CO_2_, the medium was changed with 50 μL/well RPMI-1640 containing 10% FBS. The reagent “Cell Titer 96 Aqueous One Solution Cell Proliferation” (Promega, Madison, WI, USA) was thawed for approximately 10 min in a water bath at 37 °C and 20 μL of reagent was pipetted into each well of the 96 wells plate containing the cells in 50 μL of fresh culture medium. The plate was incubated at 37 °C for 3 h in a humidified, 5% CO_2_ atmosphere. The absorbance at 490 nm was recorded using a microplate Reader (model: Synergy-H1, Bio Tek, Shoreline, WA, USA) after the 3 h incubation period.

## Additional Information

**How to cite this article:** Zeng, F. *et al*. Production of functional human nerve growth factor from the saliva of transgenic mice by using salivary glands as bioreactors. *Sci. Rep.* **7**, 41270; doi: 10.1038/srep41270 (2017).

**Publisher's note:** Springer Nature remains neutral with regard to jurisdictional claims in published maps and institutional affiliations.

## Figures and Tables

**Table 1:** Summary of TG F_0_ mice production.

Number of injected embryos	Number of transferred embryos	Number of surrogates	Number of born F_0_ mice	Number of TG F_0_ mice (transgenesis efficiency)
96	90	7	35	18 (20%)

**Table 2:** Transmission of transgene from TG F_0_ mice to their TG F_1_ offspring.

ID. of TG F_0_ mice (gender)	Copy number of transgene in TG F_0_ mice	Number of tested F_1_ mice (litter number)	Number of TG F_1_ mice (positive rate)
551 (male)	1	29 (5)	15 (51.7%)
553 (male)	1	34 (5)	16 (47.1%)
559 (female)	5	17 (2)	15 (88.2%)
560 (female)	4–5	16 (2)	14 (87.5%)

Copy number of transgene in TG F_0_ mice was determined by Southern blot as shown in Fig. 1D. Number of TG F_1_ mice was determined by PCR analysis.

**Table 3:** Primer information.

Name	Sequences (5′-3′)
P1(PCR hNGF-F)	GAACTCATATTGTACCACGACT
P2(PCR hNGF-R)	CCCCAGAATAGAATGACACC
P3(qPCR and RT-PCR hNGF-F)	GACCCAAATCCCGTTGACAGC
P4(qPCR and RT-PCR hNGF-R)	ATGGCTGGCAACTAGAAGGCACA
P5(Inverse PCR neo)	CACTTCGCCCAATAGCAG
P6(Inverse PCR PB3′TRE)	ATACAGACCGATAAAACACATG
P7(PCR Rgs7-F)	CAACCACTTACAAGAGACCCGTA
P8(PCR Rgs7-R)	GAGCCCTTAGAAATAACGTTCACC
P9(PCR EGFP-F)	TTGATGCCGTTCTTCTGCTTG
P10(PCR EGFP-R)	ACGTGCTGGTTGTTGTGCTGT
P11(PCR *PB* transposase-F)	CAGATGCCTGAGGATGGAC
P12(PCR *PB* transposase-R)	CGTTGTGGCTGTAGATGATGA
P13(RT-PCR mNGF-F)	TCCAATCCTGTTGAGAGTGGGTG
P14(RT-PCR mNGF-R)	GCCTGCTTCTCATCTGTTGTCAAC
P15(qPCR GAPDH-F)	CTCCCACTCTTCCACCTTCG
P16(qPCR GAPDH-R)	CCACCACCCTGTTGCTGTAG
